# Experience of High Tibial Osteotomy for Patients with Rheumatoid Arthritis Treated with Recent Medication: A Case Series

**DOI:** 10.3390/jcm14103332

**Published:** 2025-05-10

**Authors:** Yasuhiro Takahara, Hirotaka Nakashima, Keiichiro Nishida, Yoichiro Uchida, Hisayoshi Kato, Satoru Itani, Yuichi Iwasaki

**Affiliations:** 1 Department of Orthopedic Surgery, Nippon Kokan Fukuyama Hospital, Fukuyama 721-0927, Japan; yasuhiro_takahara@nkfh.or.jp (Y.T.); yoichiro_uchida@nkfh.or.jp (Y.U.); hisayoshi_kato@nkfh.or.jp (H.K.); satoru_itani@nkfh.or.jp (S.I.); yuichi_iwasaki@nkfh.or.jp (Y.I.); 2 Department of Orthopedic Surgery, Wakamatsu Hospital of the University of Occupational and Environmental Health, Kitakyushu 808-0024, Japan; 3 Department of Orthopedic Surgery, Okayama University Hospital, Okayama 700-8558, Japan; knishida@md.okayama-u.ac.jp

**Keywords:** high tibial osteotomy, rheumatoid arthritis, methotrexate, biologic disease-modifying antirheumatic drugs, knee surgery, joint preservation

## Abstract

**Background**: High tibial osteotomy (HTO) was generally not indicated in patients with rheumatoid arthritis (RA) because synovial inflammation may exacerbate joint damage postoperatively. Recently, joint destruction in RA has dramatically changed with the introduction of methotrexate (MTX) and biological disease-modifying antirheumatic drugs (bDMARDs). This study aimed to investigate the clinical outcomes of HTO for patients with RA treated with recent medication. **Methods**: In this study, patients with RA who underwent HTO between 2016 and 2020 were retrospectively reviewed. Patients whose follow-up period was <2 years and those whose onset of RA occurred after HTO were excluded. Clinical outcomes were investigated using the Japanese orthopedic Association (JOA) and visual analog scale (VAS) scores. **Results**: Seven patients (two males and five females, mean age 72.0 ± 6.2 years, mean body mass index 24.0 ± 2.9 kg/m^2^) were included in this study. The mean follow-up period was 62.1 ± 21.4 months. Open-wedge and hybrid closed-wedge HTO were performed in two and five cases, respectively. MTX was used for all cases. The bDMARDs were used in six cases (golimumab and tocilizumab in four and two cases, respectively). JOA scores significantly improved from 63.6 ± 10.7 preoperatively to 90.7 ± 5.3 postoperatively (*p* = 0.0167 Wilcoxon rank test). VAS scores significantly decreased from 48.6 ± 12.2 preoperatively to 11.4 ± 6.9 postoperatively (*p* = 0.017 Wilcoxon rank test). None of the patients underwent total knee arthroplasty. **Conclusions**: This study showed seven RA patients who underwent HTO treated with recent medication. The prognosis of RA, including joint destruction, has dramatically improved with induction of MTX and bDMARDs. HTO may be one of effective joint preservation surgeries even for patients with RA. To achieve the favorable outcomes, surgeons should pay attention to timing and indication of surgery.

## 1. Introduction

Biological disease-modifying antirheumatic drugs (bDMARDs) have shown attractive clinical effects on the disease activity of rheumatoid arthritis (RA) during the 15 years since their clinical application [1,2]. In addition to dramatically suppressing synovial inflammation, bDMARDs prevent joint destruction or achieve structural repair of joints in some patients.

High tibial osteotomy (HTO) has become a popular procedure for treating medial knee osteoarthritis (OA) since Coventry first reported favorable outcomes in 1979 [3]. Some modifications, such as open-wedge HTO (OWHTO) and hybrid closed-wedge HTO (hybrid CWHTO), have contributed to patient satisfaction and shortened the postoperative rehabilitation period [4,5,6,7]. However, HTO was generally not indicated in patients with RA because poor disease control may lead to the postoperative exacerbation of joint deterioration. Therefore, there were a few reports on the clinical outcomes of HTO in patients with RA. In 2019, Takahara et al. reported two patients whose RA was well controlled using bDMARDs and who underwent HTO, although this was a short-term follow-up [8]. Nakamura et al. reported a patient with RA who underwent HTO in 2023 [9]. Despite good disease control with bDMARDs, some patients show continuous inflammation of a specific joint, whereas others might already have joint destruction with irreversible functional impairment [10]. Even after amelioration of synovial inflammation with bDMARDs, joints with hyaline cartilage wear undergo osteoarthritic changes, including osteophyte formation, which can cause joint pain and restrict range of motion [11]. Thus, the purpose of this study was to report the clinical outcomes of patients with RA who underwent HTO and whose RA activity was controlled with recent medication. We hypothesized that a combination of orthopedic joint preservation surgery and medical treatment would be beneficial for patients with RA and knee arthritis.

## 2. Materials and Methods

### 2.1. Participants

Five hundred and eighty-nine patients (692 knees) who underwent HTO between January 2016 and December 2020 were retrospectively reviewed. The inclusion criteria in this study were patients with RA who were well-controlled with medication and underwent HTO. The exclusion criteria were patients with <2 years of follow-up. Patient demographics, including age, sex, body mass index (BMI), dose of MTX, and bDMARDs, were reviewed.

The indication of HTO procedure in this series were as follows: (1) medial compartment OA or osteonecrosis of the medial femoral condyle, (2) varus malalignment of the leg with persistent pain even after receiving conservative treatment for >3 months, (3) understanding that HTO is a biological procedure and a desire to undergo a joint preservation operation, and (4) agreement to perform postoperative rehabilitation. Medial compartment OA or osteonecrosis was evaluated not only using radiography but also magnetic resonance imaging. There were no age restrictions; however, patients with knee infections and severe OA of the patellofemoral (PF) joint were not indications of needing HTO. A femorotibial angle (FTA) of more than 185° (5° anatomic varus), a flexion contracture of more than 10°, or OA of the PF joint were indications for CWHTO or double-level osteotomy [12,13,14,15]. Disease activity should be controlled using medications, including bDMARDs, in patients with RA who undergo HTO. This study was approved by the local ethics committee (Nippon Kokan Fukuyama Hospital, Authorization number 2019-14). Furthermore, this study was conducted in accordance with the guidelines reported in the ethical principles for medical research involving human subjects (World Medical Association Declaration of Helsinki). Informed consent was obtained from all patients.

### 2.2. Perioperative Medication Management, Surgical Technique, and Postoperative Rehabilitation

Perioperative medication management was performed. The MTX was suspended for one week after surgery. The bDMARDs were suspended for two weeks before and after surgery.

**Figure 1:**
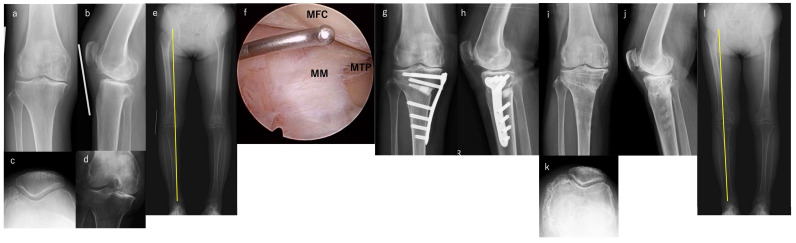
A 71-year-old female with right knee pain (Case 2). (**a**) AP view preoperatively, (**b**) lateral view preoperatively, (**c**) skyline view preoperatively, (**d**) Rosenberg view preoperatively, (**e**) full-length weight-bearing lower extremity view preoperatively, (**f**) arthroscopic findings in medial compartment, (**g**) AP view postoperatively, (**h**) lateral view postoperatively, (**i**) AP view at 5 years postoperative, (**j**) lateral view at postoperative 5 years, (**k**) skyline view at postoperative 5 years, (**l**) full-length weight-bearing lower extremity view at postoperative 5 years. Yellow line is %MA. MFC, medial femoral condyle; MM, medial meniscus; MTP, medial tibial plateau; AP, anterior–posterior; %MA, mechanical axis percentage.

A single surgeon performed all surgeries (blinded to the reviewer). Full-length weight-bearing lower extremity, anterior–posterior, lateral, and Rosenberg views were used for radiographic assessments (Figure 1a–e). Preoperative planning was performed manually using full-length weight-bearing lower extremity radiography with a picture archiving and communication system (PACS) [16,17]. Before HTO, diagnostic arthroscopy was performed in all patients (Figure 1f). Synovectomy and meniscectomy were performed as needed. OWHTO or hybrid CWHTO was performed based on the patients’ conditions, such as the degree of varus deformity, extension loss, and PF joint status (Figure 1g,h) [5,6]. Previous studies showed that the large correction in OWHTO can cause degeneration of the PF cartilage [18,19]. Thus, in cases with a correction angle of more than 12° or patellofemoral osteoarthritis, hybrid CWHTO was performed. Additionally, if there was a varus deformity in the femur, a lateral closed distal femoral osteotomy or a double-level osteotomy was performed. To balance the optimal loading with that of the historical risk of under-correction, we proposed a target weight-bearing correction between 60% and 65% [20]. The mechanical axis percentage (%MA) was determined by drawing a line from the center of the femoral head to the midpoint of the proximal talar joint surface.

OWHTO was performed according to the method described previously [21]. Artificial bone wedge, β-tricalcium phosphate (β-TCP) (Olympus Biomaterial, Tokyo, Japan), was used in the expanded portion [5]. After the osteotomy and insertion of artificial bone wedge, plate fixation was performed using a TriS locking plate (Olympus Terumo Biomaterials, Tokyo, Japan). Passive range of motion (ROM) and muscle strengthening exercises were performed on the first postoperative day. Partial weight-bearing (PWB) gait was allowed on the same day if tolerated. Full weight-bearing gait with support equipment was allowed at 4 weeks postoperatively.

Hybrid CWHTO was performed according to the method described previously [8]. After arthroscopic evaluation, a fibular osteotomy was performed obliquely at the mid portion. Subsequently, a tibial osteotomy was performed. The hinge point, which divided the proximal tibial osteotomy line by approximately 2 to 1, was then determined. After removing the lateral closed-wedge bone block, the medial side was opened, and the lateral side was closed. Plate fixation was performed using a TriS locking plate (Olympus Terumo Biomaterials, Japan). One-third PWB and ROM exercises were started on the first postoperative day. One-half PWB was started after 2 weeks postoperatively, two-third PWB 3 weeks postoperatively, and full weight bearing 4 weeks postoperatively. The patients walk with a crutch during PWB periods.

### 2.3. Clinical and Radiographic Evaluation

The Japan orthopedic Association (JOA) score, visual analog scale (VAS) score, and ROM were used to evaluate clinical outcomes [22]. Disease activity score (DAS) 28 and C-reactive protein (CRP) levels were assessed to evaluate disease activity. Major complications, including infection, peroneal nerve palsy, pulmonary embolism, delayed union, and additional surgery, including conversion to total knee arthroplasty (TKA), were also evaluated. All radiographic measurements were performed manually by one surgeon (blinded to the reviewer) using the PACS at preoperatively and final follow-up. The %MA, hip-knee-ankle angle (HKAA), and the medial proximal tibial angle (MPTA) were evaluated using full-length weight-bearing lower extremity radiography (Figure 1i–l). Anteroposterior view, Rosenberg view, lateral view, and skyline view were taken. The radiographic RA stage was evaluated based on Steinbrocker stage [23], and progression of OA was evaluated by Kellgren-Laurence (KL) grade at preoperatively and final follow-up [24].

### 2.4. Statistical Analysis

The Wilcoxon rank test was used to compare the preoperative and postoperative clinical outcomes, including the JOA score, VAS, DAS28, CRP, and ROM, and radiographic parameters, such as %MA, HKAA, and MPTA. All statistical analyses were performed using IBM SPSS version 24 (IBM Corp., Armonk, NY, USA), and statistical significance was set at *p* < 0.05.

## 3. Results

### 3.1. Patient Demographics

**Figure 2:**
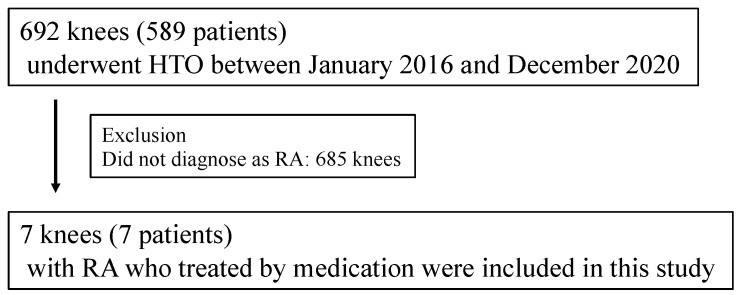
Patient selection cohort flow diagram. HTO, high tibial osteotomy; RA, rheumatoid arthritis.

Of the 589 patients (264 males and 325 females) (692 knees), 7 patients (7 knees) with RA underwent HTO in this study. All cases were followed for a minimum of 2 years (follow-up rate 100%). Thus, seven patients (two males and five females, mean age 72.0 ± 6.2 years; mean BMI 24.0 ± 2.9 kg/m^2^) were included in this study (Figure 2). The mean follow-up period was 62.1 ± 21.4 months, and OWHTO and hybrid CWHTO were performed in two and five cases, respectively. The mean dose of MTX was 7.7 ± 2.4 mg/week. The bDMARDs used were in six cases (golimumab (GOL) and tocilizumab (TCZ) in four and two cases, respectively) (Table 1). Diabetes mellitus (DM) was present in four cases and hypertension was in one case. All co-morbidities were treated by internal medicine physicians.

### 3.2. Clinical Outcomes

The clinical outcomes of each patient are presented in Table 2. The JOA score was significantly improved from 63.6 ± 10.7 preoperatively to 90.7 ± 5.3 postoperatively (*p* = 0.016, Wilcoxon rank test). The VAS score also significantly decreased from 48.6 ± 12.2 preoperatively to 11.4 ± 6.9 postoperatively (*p* = 0.016, Wilcoxon rank test). DAS28 significantly improved from 3.0 ± 0.2 preoperatively to 1.6 ± 0.5 postoperatively (*p* = 0.018, Wilcoxon rank test). Although CRP improved from 1.0 ± 1.0 mg/dL preoperatively to 0.3 ± 0.3 mg/dL postoperatively, no significant difference was found in CRP (*p* = 0.063, Wilcoxon rank test). The extension angle significantly improved from −7.9 ± 4.9° preoperatively to 0.0 ± 0.0° postoperatively (*p* = 0.026, Wilcoxon rank test). However, the flexion angle did not significantly improve, from 126.4 ± 10.7° preoperatively to 128.6 ± 11.1° postoperatively (*p* = 0.480, Wilcoxon rank test). Hardware removal (HWR) was performed in all cases after bone union. No infection, delayed union, or other complications were observed. None of the patients underwent a TKA.

### 3.3. Radiographic Outcomes

Preoperative Steinbrocker stage and KL grade were described in Table 3. At final follow-up, OA progression was observed in 2 cases according to KL grade evaluation. The %MA significantly increased from 26.1 ± 9.0 preoperatively to 59.6 ± 4.2 postoperatively (*p* = 0.018, Wilcoxon rank test). HKAA significantly decreased from 5.9 ± 2.6 varus preoperatively to 2.9 ± 1.5 valgus postoperatively (*p* = 0.018, Wilcoxon rank test). MPTA significantly increased from 84.9 ± 1.1 preoperatively to 92.5 ± 4.2 postoperatively (*p* = 0.028, Wilcoxon rank test).

## 4. Discussion

This study showed seven RA patients who underwent HTO treated with recent medication. Joint replacement surgery, including TKA, is a common procedure for patients with RA [25,26,27,28]. However, if the suppression of joint deterioration is possible with medications, such as MTX and bDMARDs, a more challenging surgical procedure may be considered to further improve patient function, demand, and satisfaction. The paradigm of surgical reconstruction of the rheumatoid forefoot, which was previously typified by the earlier Lelièvre procedure, has shifted to joint preservation surgery [29,30,31]. Thus, joint preservation surgery may be effective for the knee joint if knee joint deterioration can be suppressed using bDMARDs. In fact, arthroscopic synovectomy of the knee remains an effective treatment [32,33,34]. Moreover, joint preservation surgery has been performed not only on the forefoot but also on the hip and shoulder joints [35,36,37].

Although around-the-knee osteotomy (AKO) is an effective treatment for medial compartment OA, there are few reports on AKO in patients with RA. Ahlberg et al. reported the outcomes in 11 patients with RA in 1968 [38]. Two and nine cases were tibial and distal femur osteotomies, respectively. According to their study, 20% of the patients improved, and 80% remained unimproved (one case was not evaluated). Chan et al. showed outcomes of HTO in 36 patients with RA in 1978 [39]. They reported that 42%, 19%, and 39% were good, satisfactory, and poor at 1–6-year postoperative follow-up, respectively. Despite these favorable results, the prevalence of “poor” cases increased 3 years postoperatively, and the performance of HTO for RA was reported to be inferior to that of HTO for OA symptoms. In 1969, Benjamin reported that 81% (17/21) of the patients who underwent double osteotomy for a painful rheumatoid knee had improved [40]. Iveson et al. conducted a comparative study of single and double osteotomies for OA and RA [41]. They reported that 67% and 68% of patients with RA who underwent single and double osteotomies improved, respectively. However, in 1987, Schüller et al. reported poor results with double osteotomy for rheumatoid knees [42]. These results were obtained before the use of antirheumatoid drugs, including bDMARDs.

Recent studies have shown that the radiographic characteristics of patients with RA change after bDMARD induction. Takeda et al. described that the percentage of ‘‘OA-like RA’’ had a significant increasing trend from 20.9% in 2006 to 67.7% in 2020 in patients who underwent TKA [43]. Fujimura et al. reported that following the introduction of bDMARDs, typical radiographic findings of rheumatoid knees decreased, and secondary OA-like changes, characterized by osteophyte formation and medial-dominant joint space narrowing, increased in the knees of TKA recipients [44]. Takeda et al. reported that the radiographs of TKA recipients with RA have increasingly presented with OA features recently [45]. These studies suggested that advances in RA treatment suspended the progression of RA. Recently, a case where a patient with RA underwent HTO has been reported [8,9]. Koga et al. also described a patient with psoriatic arthritis who underwent HTO in 2025 [46]. These reports had short-term follow-up. Thus, to our knowledge, this is one of the few studies reporting mid-term outcomes of HTO in RA patients with bDMARDs therapy. Advances in RA treatment may expand the indications for HTO even in patients with RA and rheumatic diseases. Moreover, Deckey et al. reported the clinical outcomes of patients with RA who underwent unicompartmental knee arthroplasty. There was no significant difference in conversion to TKA between patients who had and did not have RA. Their results may indicate that modern management of RA could allow for expanded UKA indications for patients with RA [47].

Because HTO is a biological procedure, OA progression is inevitable over a longer follow-up period. Recent studies have shown inferior clinical outcomes of OWHTO in patients with severe OA compared with those in patients with early OA. Nha described that the KL grade was significantly negatively correlated with the postoperative Oxford Knee Score (OKS) [48]. Takahara et al. reported that the clinical score of KL 3 was lower than that of KL1 and 2 [21]. Also, KL 3 was a risk factor for radiological OA progression according to their study [21]. Thus, HTO may be recommended in early radiological stages, even in patients with RA. In addition, a recent study reported by Herbst et al. showed that overweight patients benefit from HTO to the same extent as patients with normal weights, but showed inferior mid-term results [49]. Thus, BMI may be an important factor in obtaining good outcomes. In fact, the BMI in this case series was normal. This may be one of the reasons for the good clinical outcomes in this study.

Hardware was removed in all cases in this study. A recent study by Katayama et al. demonstrated the effect of HWR after OWHTO [50]. They described that HWR improves the flexion angle, clinical outcomes, and hardware-related complications after OWHTO. In addition, Sidhu et al. reported high rates of HWR after OWHTO [51]. Although the patients in this study were older (mean age 72.0 ± 6.2 years), HWR may be useful to improve the clinical outcomes.

This study had some limitations. First, it was a retrospective case series with a small number of patients and mid-term postoperative observation periods. Thus, a large number of patients and long-term follow-ups are needed to confirm the clinical outcomes. Additionally, prospective comparative studies are warranted. Second, the study was conducted at a single hospital, and all surgeries were performed by a single surgeon. Therefore, the senior author’s expertise may have introduced a bias. Third, only the JOA and VAS scores were used to evaluate the clinical outcomes. Patient-reported outcome measures such as the Knee Injury and Osteoarthritis Outcome Score, OKS, and Short Form 36 were not used.

## 5. Conclusions

This study showed seven RA patients who underwent HTO treated with recent medication. The prognosis of RA, including joint destruction, has dramatically improved with the induction of MTX and bDMARDs. HTO may be one of effective joint preservation surgeries even for patients with RA. To achieve the favorable outcomes, surgeons should pay attention to timing and indication of surgery.

## Figures and Tables

**Table 1:** Patient demographics.

Case	Age (Year)	Sex	BMI (kg/m^2^)	Follow-Up (Months)	MTX (mg/Week)	bDMARDs	Co-Morbidity
1	82	Male	23.5	24	10	TCZ → GOL	DM
2	71	Female	22.7	60	10	TCZ	None
3	67	Female	29.1	77	4	GOL	None
4	75	Female	22.3	84	8	GOL	None
5	67	Female	22.9	82	6	GOL	DM
6	77	Female	20.6	60	6	TCZ	DM, HT
7	65	Male	26.8	48	10		None

BMI, body mass index; MTX, methotrexate; bDMARDs, biological disease-modifying antirheumatic drugs; TCZ, tocilizumab; GOL, golimumab; DM, diabetes mellitus; HT, hypertension.

**Table 2:** Clinical outcomes.

Case	HTO	Pre JOA	Post JOA	Pre VAS	Post VAS	Pre DAS28	Post DAS28	Pre CRP (mg/dL)	Post CRP (mg/dL)	Pre Ext (°)	Post Ext (°)	Pre Flex (°)	Post Flex (°)
1	OWHTO	70	95	70	20	3.01	1.38	0.32	0.05	0	0	130	135
2	OWHTO	80	90	40	20	2.8	2.6	0.25	0.16	−5	0	120	120
3	Hybrid CWHTO	50	90	40	10	3.1	1.3	1.0	0.32	−10	0	135	130
4	Hybrid CWHTO	55	95	40	10	3.3	1.3	3.08	0.08	−15	0	130	145
5	Hybrid CWHTO	55	80	40	10	3.04	1.24	0.96	0.05	−10	0	105	110
6	Hybrid CWHTO	65	90	60	10	2.92	1.92	0.96	0.88	−10	0	135	130
7	Hybrid CWHTO	70	95	50	0	2.99	1.56	0.3	0.43	−5	0	130	130

HTO, high tibial osteotomy; JOA, Japan orthopedic Association; VAS, visual analog scale; DAS, disease activity score; CRP, C-reactive protein; OWHTO, open-wedge high tibial osteotomy; hybrid CWHTO, hybrid closed-wedge high tibial osteotomy.

**Table 3:** Radiographic outcomes.

Case	Preoperative Steinbrocker Stage	Preoperative KL Grade	Postoperative KL Grade
1	Ⅱ	2	2
2	Ⅱ	2	3
3	Ⅱ	3	3
4	Ⅰ	1	1
5	Ⅱ	3	4
6	Ⅱ	3	3
7	Ⅱ	3	3

KL, Kellgren–Lawrence.

## Data Availability

The data of this study are included within the article and available from the corresponding author on reasonable request.

## Abbreviations

HTO, high tibial osteotomy; RA, rheumatoid arthritis; MTX, methotrexate; bDMARDs, biological disease-modifying antirheumatic drugs; JOA, Japan orthopedic Association; VAS, visual analog scale; OA, osteoarthritis; OWHTO, open-wedge high tibial osteotomy; hybrid CWHTO, hybrid closed-wedge high tibial osteotomy; BMI, body mass index; PF, patellofemoral; FTA, femorotibial angle; PACS, archiving and communication system; %MA, mechanical axis percentage; ROM, range of motion; PWB, partial weight bearing; DAS, disease activity score; CRP, C-reactive protein; TKA, total knee arthroplasty; HKAA; hip-knee-ankle angle, KL, Kellgren–Lawrence; MFC, medial femoral condyle, MM, medial meniscus; MTP, medial tibial plateau; AP, anterior–posterior MPTA, medial proximal tibial angle; GOL; golimumab, TCZ, tocilizumab; DM, diabetes mellitus; PtGA, patients’ global assessment of health; HWR; Hardware removal, AKO, around-the-knee osteotomy; OKS, Oxford Knee Score.
